# Supplementary material for: Modeling glioblastoma heterogeneity as a dynamic network of cell states
Source: Mol Syst Biol. 2021 Sep 16;17(9):e10105. doi: 10.15252/msb.202010105 (PMC8444284; doi:10.15252/msb.202010105)
Supplement: Supplementary file 5 — Source Data for Figure 3 [file MSB-17-e10105-s001.zip › Figure3A_sourcedata/GSEA_3065/nosigdb_state1.GseaPreranked.1622718409535/RICHARDS_NEFTEL_CELL_2019_MES2.html]

Details for gene set RICHARDS\_NEFTEL\_CELL\_2019\_MES2[GSEA]

|  || Dataset | state1 |
| Phenotype | NoPhenotypeAvailable |
| Upregulated in class | na\_pos |
| GeneSet | RICHARDS\_NEFTEL\_CELL\_2019\_MES2 |
| Enrichment Score (ES) | 0.5765423 |
| Normalized Enrichment Score (NES) | 1.7957681 |
| Nominal p-value | 0.0 |
| FDR q-value | 0.0020729243 |
| FWER p-Value | 0.048 |
Table: GSEA Results Summary

  

Fig 1: Enrichment plot: RICHARDS\_NEFTEL\_CELL\_2019\_MES2      
 Profile of the Running ES Score & Positions of GeneSet Members on the Rank Ordered List

  

| PROBE | GENE SYMBOL | GENE\_TITLE | RANK IN GENE LIST | RANK METRIC SCORE | RUNNING ES | CORE ENRICHMENT || 1 | IGFBP3 |  |  | 15 | 0.664 | 0.1009 | Yes |
| 2 | ANXA2 |  |  | 48 | 0.482 | 0.1719 | Yes |
| 3 | LDHA |  |  | 80 | 0.394 | 0.2295 | Yes |
| 4 | LGALS3 |  |  | 117 | 0.348 | 0.2796 | Yes |
| 5 | FTL |  |  | 134 | 0.333 | 0.3293 | Yes |
| 6 | VIM |  |  | 175 | 0.308 | 0.3726 | Yes |
| 7 | SLC2A1 |  |  | 235 | 0.283 | 0.4102 | Yes |
| 8 | CDKN1A |  |  | 262 | 0.274 | 0.4499 | Yes |
| 9 | BNIP3 |  |  | 570 | 0.190 | 0.4480 | Yes |
| 10 | PFKP |  |  | 594 | 0.187 | 0.4744 | Yes |
| 11 | AKAP12 |  |  | 605 | 0.185 | 0.5020 | Yes |
| 12 | RPL21 |  |  | 660 | 0.177 | 0.5237 | Yes |
| 13 | SQSTM1 |  |  | 701 | 0.172 | 0.5462 | Yes |
| 14 | NDRG1 |  |  | 765 | 0.163 | 0.5649 | Yes |
| 15 | PGK1 |  |  | 875 | 0.148 | 0.5765 | Yes |
| 16 | SERTAD1 |  |  | 1327 | 0.101 | 0.5462 | No |
| 17 | NAMPT |  |  | 1622 | 0.081 | 0.5288 | No |
| 18 | UFM1 |  |  | 1663 | 0.078 | 0.5367 | No |
| 19 | GBE1 |  |  | 1769 | 0.071 | 0.5370 | No |
| 20 | HSPA9 |  |  | 1793 | 0.070 | 0.5455 | No |
| 21 | SLC6A6 |  |  | 1856 | 0.067 | 0.5495 | No |
| 22 | DDIT3 |  |  | 2008 | 0.059 | 0.5433 | No |
| 23 | WARS |  |  | 2293 | 0.048 | 0.5218 | No |
| 24 | ADM |  |  | 2325 | 0.046 | 0.5257 | No |
| 25 | HERPUD1 |  |  | 2598 | 0.036 | 0.5037 | No |
| 26 | ATF3 |  |  | 2855 | 0.029 | 0.4821 | No |
| 27 | ENO2 |  |  | 3161 | 0.021 | 0.4544 | No |
| 28 | PPP1R15A |  |  | 3296 | 0.018 | 0.4436 | No |
| 29 | EPAS1 |  |  | 3624 | 0.011 | 0.4120 | No |
| 30 | XPOT |  |  | 5007 | -0.013 | 0.2735 | No |
| 31 | NRN1 |  |  | 5146 | -0.015 | 0.2617 | No |
| 32 | ASNS |  |  | 5370 | -0.018 | 0.2418 | No |
| 33 | SLC39A14 |  |  | 5952 | -0.028 | 0.1871 | No |
| 34 | MT1X |  |  | 6479 | -0.038 | 0.1395 | No |
| 35 | DNAJB9 |  |  | 7191 | -0.055 | 0.0758 | No |
| 36 | GOLT1B |  |  | 7281 | -0.057 | 0.0756 | No |
| 37 | INSIG2 |  |  | 7941 | -0.079 | 0.0207 | No |
| 38 | HILPDA |  |  | 8612 | -0.112 | -0.0301 | No |
| 39 | TRAM1 |  |  | 8618 | -0.112 | -0.0132 | No |
| 40 | GDF15 |  |  | 8679 | -0.116 | -0.0015 | No |
| 41 | BNIP3L |  |  | 8707 | -0.118 | 0.0140 | No |
| 42 | P4HA1 |  |  | 8810 | -0.125 | 0.0229 | No |
| 43 | SLC2A3 |  |  | 9156 | -0.159 | 0.0124 | No |
| 44 | PLOD2 |  |  | 9169 | -0.161 | 0.0361 | No |
| 45 | HSPA5 |  |  | 9533 | -0.234 | 0.0352 | No |
Table: GSEA details [plain text format]

  

Fig 2: RICHARDS\_NEFTEL\_CELL\_2019\_MES2: Random ES distribution      
 Gene set null distribution of ES for **RICHARDS\_NEFTEL\_CELL\_2019\_MES2**
